# Retrospective Serological Survey of Equine Influenza Virus and Piroplasm Parasites in Free-Ranging Wild Equids from Kenya and Tanzania

**DOI:** 10.3390/vetsci13101034

**Published:** 2026-09-30

**Authors:** Silvia Marchi, Charles Issel, Paul Kanyari, Julius Keyyu, Pompei Bolfa

**Affiliations:** 1 Department of Biomedical Sciences, Ross University School of Veterinary Medicine, West Farm, St. Kitts, Basseterre KN0101, Saint Kitts and Nevis; 2 Department of Veterinary Science, Gluck Equine Research Center, University of Kentucky, Lexington, KY 40546-0099, USA; 3 Department of Pathology, Microbiology and Parasitology, Faculty of Veterinary Medicine, College of Agriculture and Veterinary Sciences, University of Nairobi, Nairobi P.O. Box 29053, Kenya; 4 Tanzania Wildlife Research Institute, Arusha P.O. Box 661, Tanzania

**Keywords:** zebra serology, equine influenza, equine infectious anemia, equine piroplasmosis, vector borne diseases

## Abstract

Wild equids may act as reservoirs for infectious pathogens affecting domestic equine populations, yet data on exposure to equine infectious anemia virus (EIAV), equine influenza virus (EIV), and equine piroplasmosis agents in African wildlife remain scarce. Our objectives were to retrospectively determine serological evidence of exposure to EIAV, EIV, *Theileria equi*, and *Babesia caballi* among Grevy’s zebras and rhinoceroses from Kenya and Tanzania. A total of 284 archived serum samples from 215 Grevy’s zebras and 69 rhinoceroses were analyzed using commercial ELISAs. Positive EIAV screening tests were retested using a second confirmatory assay. No confirmed EIAV antibodies were detected in zebras or rhinoceroses. Antibodies against EIV were identified in 20/215 zebras (9.3%), all originating from Serengeti National Park. *Theileria equi* antibodies were detected in 194/206 zebras (94.2%), while *Babesia caballi* antibodies were detected in 8/206 zebras (3.9%). Rhinoceroses were negative for all pathogens tested. This study provides evidence of widespread exposure of Grevy’s zebras to *T. equi* and limited exposure to EIV in East Africa while failing to identify serologic evidence of EIAV infection in zebras or rhinoceroses. These findings improve understanding of infectious disease ecology at the wildlife–livestock interface and support continued wildlife surveillance programs.

## Simple Summary

Wild animals that live alongside domestic horses, donkeys, and other equids can sometimes carry infectious diseases that affect animal health, conservation programs, and livestock production. However, little is known about the exposure of free-ranging zebras and rhinoceroses in East Africa to several important equine diseases. The aim of this study was to investigate evidence of previous exposure to four pathogens in archived blood samples collected from Grevy’s zebras and rhinoceroses in Kenya and Tanzania. A total of 284 samples from 215 zebras and 69 rhinoceroses were tested for antibodies against the virus that causes equine infectious anemia, the virus responsible for equine influenza, and two tick-borne blood parasites that cause equine piroplasmosis. No evidence of exposure to equine infectious anemia was detected in either species. Exposure to equine influenza was identified in a small proportion of zebras, all originating from the Serengeti ecosystem in Tanzania. Exposure to one of the tick-borne parasites was extremely common in zebras, while exposure to the second parasite was uncommon. All rhinoceroses tested negative for all pathogens evaluated. These findings suggest that free-ranging Grevy’s zebras are frequently exposed to certain tick-transmitted parasites and occasionally exposed to influenza viruses, whereas exposure to the other pathogens studied appears limited or absent in the sampled animals. The study provides valuable baseline information on disease exposure in wildlife and contributes to a better understanding of pathogen circulation at the interface between wildlife and domestic animals. Such information can support wildlife conservation efforts, animal health surveillance, and future disease monitoring programs in East Africa.

## 1. Introduction

Infectious diseases occurring at the wildlife–livestock interface represent an important challenge for animal health, livestock production, and wildlife conservation. Free-ranging wildlife populations may serve as reservoirs, maintenance hosts, or sentinels for pathogens affecting domestic animals, while increasing contact between wildlife and livestock can facilitate pathogen transmission in both directions. Consequently, surveillance of wildlife populations contributes to understanding pathogen circulation, evaluating risks of spillover events, and informing disease management strategies in shared ecosystems [1,2].

In East Africa, wild and domestic equids frequently coexist across extensive rangelands where opportunities for direct and indirect pathogen transmission are common. Grevy’s zebra (*Equus grevyi*), plains zebra (*Equus quagga*), donkeys, and horses often share grazing areas and water sources, creating ecological conditions favorable for exposure to infectious agents affecting equids. Despite this close overlap, information regarding exposure of free-ranging zebras to several important equine pathogens remains limited [3].

The Grevy’s zebra is of particular interest because it is classified as Endangered by the International Union for Conservation of Nature (IUCN), making disease surveillance relevant not only from a veterinary perspective but also for conservation management [4]. Declining wildlife populations may be particularly vulnerable to the impacts of emerging and endemic infectious diseases, and baseline information on pathogen exposure is essential for evaluating population health and future disease risks [5].

Equine infectious anemia (EIA) is a globally distributed viral disease of equids caused by equine infectious anemia virus (EIAV), a lentivirus that establishes lifelong infection in affected animals. The disease is primarily transmitted through blood-feeding insects and contaminated instruments, and infected animals may remain long-term carriers. Because of its economic importance and potential impact on equine movement and trade, EIA is a disease listed by the World Organisation for Animal Health (WOAH) and remains subject to extensive surveillance programs worldwide [6,7]. Evidence regarding the role of free-ranging zebras and rhinoceroses in EIAV epidemiology remains scarce, and data from East Africa are particularly limited [8].

Equine influenza (EI) is one of the most contagious respiratory diseases of equids and is caused primarily by influenza A H3N8 viruses. Although infection has been extensively investigated in horses, relatively little information is available regarding exposure among free-ranging wild equids [9]. Serological evidence of exposure in wildlife populations may provide insight into pathogen circulation and possible interactions between domestic and wild equid populations.

Equine piroplasmosis is a tick-borne disease caused mainly by the intraerythrocytic protozoa *Theileria equi* and *Babesia caballi.* The disease is globally distributed in tropical and subtropical regions and represents a significant health and trade concern for equids. In endemic regions, infected animals frequently become chronic carriers and may serve as long-term reservoirs for infection. Recent reviews emphasize that *T. equi* is generally more persistent and more widely distributed than *B. caballi*, resulting in higher seroprevalence in many endemic areas. Nevertheless, information regarding exposure among free-ranging zebras and rhinoceroses in East Africa remains fragmented [10,11,12,13].

The objective of this study was therefore to investigate serological evidence of exposure to EIAV, equine influenza virus (EIV), *Theileria equi,* and *Babesia caballi* in free-ranging zebras and rhinoceroses sampled in Kenya and Tanzania. By evaluating multiple pathogens simultaneously, this study provides baseline data relevant to wildlife health, conservation, and disease surveillance at the wildlife–livestock interface in East Africa.

## 2. Materials and Methods

### 2.1. Study Plan

Bio-banked serum and plasma samples were examined in this project, which were previously collected from African National parks and National reserves under each country’s regulations. Samples originated from an archived wildlife serum bank and were collected prior to 2014. Exact collection dates were unavailable for many samples. Consequently, analyses were conducted using the archived specimens as a cross-sectional convenience sample representative of historical pathogen exposure rather than a defined temporal survey.

A total of 284 animals were tested for equine infectious anemia (EIAV), equine influenza (EI), and equine piroplasmosis (*Theileria equi* and *Babesia caballi*), 3 of the 11 WOAH notifiable diseases in equines. The animals investigated for seroprevalence were N = 215 Grevy’s zebras (wild equids) and N = 69 rhinoceroses, including black rhinoceroses (*Diceros bicornis*) and white rhinoceroses (*Ceratotherium simum*). From these and the available records, there were 42 samples fromblack rhinoceroses, 6 samples from white rhinoceroses and 21 samples for which the species of rhinoceroses was not available. Because species-level records were incomplete within the archived database, results for rhinoceroses were analyzed collectively. All tested animals were unvaccinated and clinically healthy.

Species-specific counts for some archived samples were unavailable and therefore location summaries were reported at the level available in the source records. They originated from 8 locations in Kenya and 3 locations in Tanzania plus 25 samples with unknown origin except the country (Supplemental Table S1). N = 201 samples originated from 3 areas of Tanzania, most of them, n = 185, from Serengeti National Park and N = 83 were gathered from 13 known Kenyan regions (Figure 1). From the Tanzanian locations N = 185 zebra and N = 16 rhinos were screened, while Kenya provided N = 30 zebra and N = 53 rhinos. Out of the 284 animals, N = 135 were females and N = 149 were males.

Tanzanian tests were conducted at the Tanzania Wildlife Research Institute (TAWIRI), a parastatal organization under the Ministry of Natural Resources and Tourism responsible for conducting and coordinating wildlife research in the United Republic of Tanzania, Njiro Hill, Arusha, while Kenyan samples were tested at the Department of Pathology, Microbiology and Parasitology, Faculty of Veterinary Medicine, College of Agriculture and Veterinary Sciences, University of Nairobi, Kenya.

### 2.2. Serological Analyses

Five commercially available ELISA kits were used. Antibodies against EIAV were screened using a commercial competitive ELISA (IDEXX Laboratories, Montpellier, France). Reactive samples were retested using a second ELISA (VMRD, Pullman, Washington, DC, USA) according to WOAH recommendations for confirmation of EIAV serologic results. Antibodies against influenza A virus were detected using a blocking ELISA (IDvet, Grabels, France). Antibodies against *T. equi* and *B. caballi* were detected using species-independent competitive ELISAs (VMRD, Pullman, Washington, DC, USA). All assays were performed according to manufacturers’ instructions, with all samples tested in duplicate and positive samples were retested. Generative AI tools were used in the preparation of this manuscript.

### 2.3. Statistical Analysis

Apparent seroprevalence was calculated as the proportion of seropositive animals among animals tested. Exact binomial 95% confidence intervals (Clopper-Pearson method) were calculated for all prevalence estimates. Comparisons of seroprevalence between countries and host species were performed using Fisher’s exact test because several contingency table cells contained small expected counts. Statistical analyses were conducted using a two-sided significance level of α = 0.05.

## 3. Results

The overall serology results, are summarized in Table 1 with a detailed geographical distribution of samples and their corresponding results in Supplemental Table S1.

Zebras showed no serological evidence of previous exposure to EIAV, while seroconversion was detected for EI and piroplasmosis. Twenty zebra samples which were initially reactive on the EIAV screening were classified as false positives on confirmatory testing, resulting in no confirmed EIAV-seropositive animals among the 284 samples tested.

Antibodies against EIV were detected in 20 of 215 zebras (9.3%; 95% CI: 5.8–13.9%). All EIV-positive samples originated from Serengeti National Park, Tanzania. No rhinoceroses were seropositive for EIV.

Antibodies against *T. equi* were detected in 194 of 206 zebras (94.2%; 95% CI: 89.9–97.0%), while antibodies against *B. caballi* were detected in 8 of 206 zebras (3.9%; 95% CI: 1.7–7.5%). *T. equi*-positive animals were identified in all sampled regions, whereas *B. caballi*-positive animals were detected only in Serengeti National Park and Ngorongoro Crater.

All rhinoceroses tested negative for antibodies against EIAV, EIV, *T. equi,* and *B. caballi*.

## 4. Discussion

This survey found no serological evidence of EIAV exposure in free-ranging zebras or rhinoceroses from Kenya and Tanzania. In contrast, exposure to EIV and particularly *T. equi* was documented in zebras, while all rhinoceroses remained seronegative for all pathogens evaluated. The observed distribution reflects sample availability within this historical convenience sample and should not be interpreted as representative of the broader populations across the study region.

Interpretation of geographic patterns should also be undertaken cautiously because sampling intensity differed substantially among locations. Most specimens originated from Serengeti National Park (185/284), whereas several locations were represented by comparatively few samples. Consequently, the probability of detecting low-prevalence pathogen exposure was considerably greater in Serengeti than in sparsely sampled regions. Absence of seropositive animals at locations with small sample sizes therefore should not be interpreted as definitive evidence of pathogen absence. Although samples were collected during different seasons, detailed seasonal metadata were unavailable for many archived specimens, preventing formal analysis of seasonal patterns. Seasonal variation in vector abundance and pathogen transmission may influence exposure to influenza viruses and tick-borne pathogens, and future prospective studies with precise sampling dates would be useful for evaluating such effects.

No rhinoceroses were seropositive for any pathogen evaluated. However, interpretation should be cautious because sample numbers were relatively small compared with the number of zebra samples and because assay performance has not been extensively validated in rhinoceroses species. Previous serosurveys have demonstrated exposure of African rhinoceroses to other infectious agents, indicating that negative serologic findings do not necessarily imply absence of pathogen exposure risk [14,15,16].

The lack of detectable EIAV antibodies suggests that EIAV was not widely circulating among the sampled animals during the study period. This finding aligns with the limited available evidence indicating a low occurrence of EIAV exposure in free-ranging African zebras. Nevertheless, the retrospective nature of the study and the uneven geographic distribution of samples limit broader epidemiological inference. Future studies incorporating both serological and molecular approaches are warranted to clarify the role of free-ranging African equids in the ecology and transmission dynamics of EIAV [8,17].

Detection of EIV antibodies in 9.3% of zebras suggests previous exposure to influenza A viruses within the Serengeti ecosystem. Because all seropositive animals originated from Serengeti National Park, exposure may occur through localized circulation or sporadic spillover events involving domestic equids. Similar information remains scarce for free-ranging zebra populations, making these findings an important contribution to understanding equine influenza ecology in East Africa [9].

The high prevalence of *T. equi* antibodies observed here is consistent with molecular evidence reported by Hawkins et al. (2015), who detected *T. equi* DNA in 100% (16/16) of Grevy’s zebras sampled in northern Kenya using nested PCR. Together, these molecular and serological findings support the hypothesis that *T. equi* is endemic in East African zebra populations and that free-ranging zebras may contribute to long-term maintenance of the parasite within local transmission cycles. In contrast, *B. caballi* exposure remained uncommon, consistent with global studies showing lower prevalence and reduced persistence of *B. caballi* infection compared with *T. equi* [3,11,13].

Limitations of our study include the absence of precise collection dates precluded assessment of temporal trends in pathogen exposure. Several limitations should also be considered when interpreting these findings. First, serological assays detect evidence of previous exposure rather than active infection. Antibody detection does not distinguish among recent infection, historical exposure, or persistent infection, and seronegative animals may still be infected if sampled before seroconversion or after declining antibody titers. Consequently, seroprevalence estimates should be interpreted as indicators of prior pathogen exposure rather than direct evidence of current infection. Molecular approaches such as PCR identify pathogen nucleic acids and are more appropriate for detecting active infections, as demonstrated in Grevy’s zebras where *T. equi* infection is reported using PCR-based methods [3]. A further limitation concerns assay validation. The commercial competitive ELISAs used in this study were developed primarily for domestic animal species (ID.vet and VMRD). Competitive and blocking ELISAs are generally less dependent on species-specific conjugates and therefore can be applied across multiple taxa. However, formal validation data for Grevy’s zebras and African rhinoceroses are limited or unavailable. Consequently, although these assays have been widely used for surveillance purposes, diagnostic sensitivity and specificity in the wildlife species examined herein remain uncertain and could have influenced prevalence estimates [18].

Another limitation relates to the use of archived serum samples. The duration and conditions of storage varied among specimens, and precise storage histories were unavailable. Antibodies are generally stable during long-term frozen storage, particularly when repeated freeze–thaw cycles are minimized, but degradation cannot be completely excluded, and they might differ in long-term stored wildlife sera. Therefore, some degree of misclassification due to altered antibody detectability is possible, although the overall impact is likely limited [19].

These findings provide baseline surveillance data for important equine pathogens in free-ranging wildlife and reinforce the value of continued disease monitoring at the wildlife–livestock interface in East Africa.

## Figures and Tables

**Figure 1:**
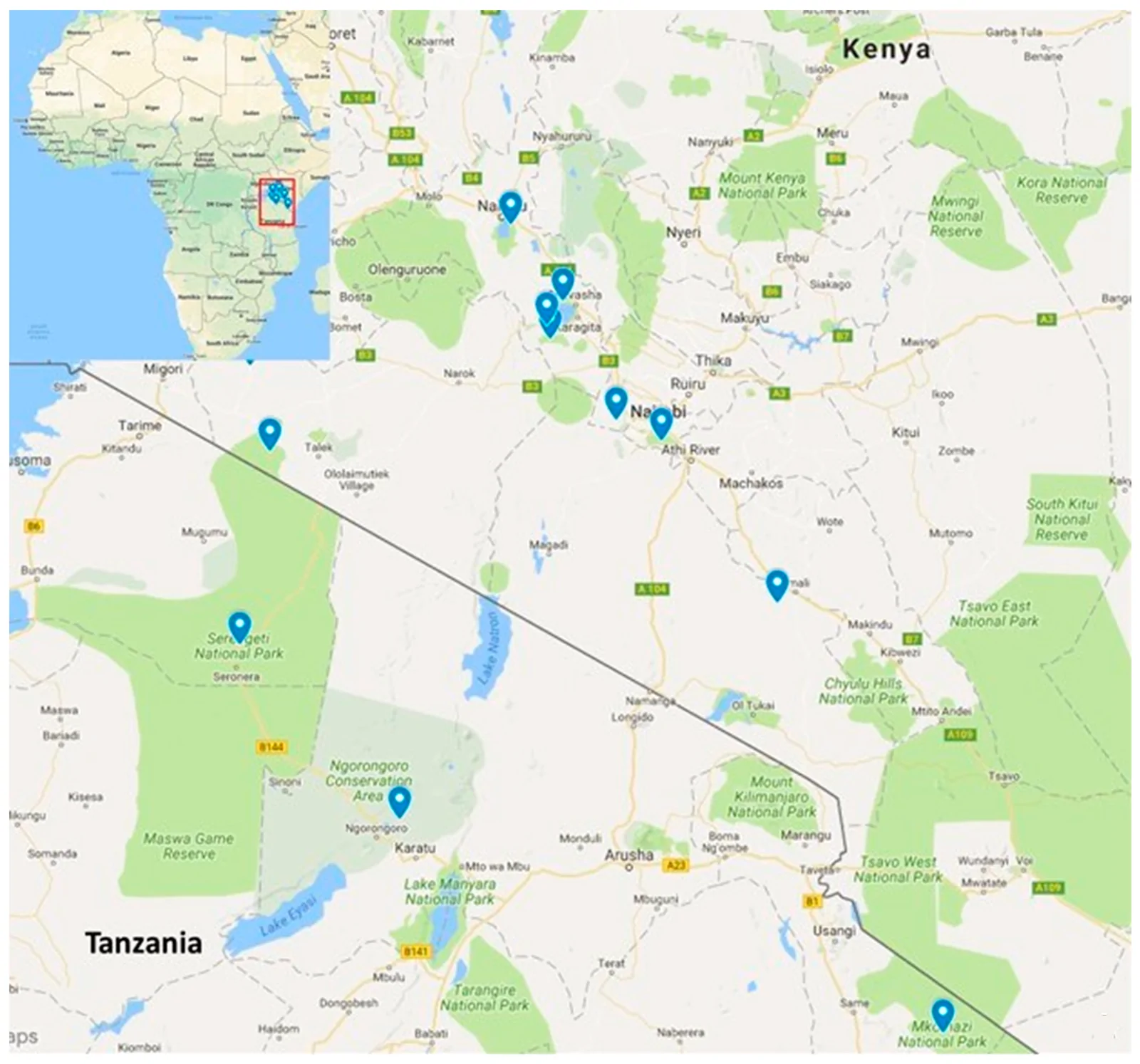
Serum samples of wild equids and rhinoceroses originated mainly from 11 different locations in Kenya and Tanzania.

**Table 1:** Serological evidence of exposure to equine infectious anemia virus (EIAV), equine influenza virus (EIV), *Theileria equi*, and *Babesia caballi* in free-ranging zebras and rhinoceroses from Kenya and Tanzania.

Species	N Tested	EIAV	EIV	*T. equi* *	*B. caballi* *
Zebra	215	0/215 (0.0%)	20/215 (9.3%)	194/206 (94.2%)	8/206 (3.9%)
Rhinoceroses	69	0/69 (0.0%)	0 (0.0%)	0/61 (0.0%)	0/61 (0.0%)
Total	284	0/284 (0.0%)	20/284 (7.0%)	194/267 (72.7%)	8/267 (3.0%)

* Insufficient serum volume available for testing in select individuals.

## Data Availability

The original contributions presented in this study are included in the article/Supplementary Material. Further inquiries can be directed to the corresponding author.

## Supplementary Materials

The following supporting information can be downloaded at: https://www.mdpi.com/article/10.3390/vetsci13101034/s1. Table S1: Species distribution, sampling locations, and serological findings of study animals.
