# Supplementary material for: Retrospective Serological Survey of Equine Influenza Virus and Piroplasm Parasites in Free-Ranging Wild Equids from Kenya and Tanzania
Source: Vet Sci. 2026 Sep 30;13(10):1034. doi: 10.3390/vetsci13101034 (PMC13709856; doi:10.3390/vetsci13101034)
Supplement: Supplementary file 1 [file vetsci-13-01034-s001.zip › vetsci-4563712-supplementary.pdf]

Supplemental Table S1: Species distribution, sampling locations, and serological findings of study animals.

Disease name	B. caballi	T. equi	EIA	EIA retest VMRD	Influenza
Total animals tested	**267**	**267**	**284**	**20**	**284**
Zebras (number tested)	206	206	215	20	215
Rhinoceroses (number tested)	61	61	69	0	69
Totals positives (number)	8	194	16	0	20
Totals positives (%)	**3.0**	**72.7**	**5.6**	**0.0**	**7.0**
Zebras positive samples (number from total)	8	194	16	0	20
Rhinoceroses positive samples (number from total)	0	0	0	0	0
Zebras percentage positive samples from total (%)	3.0%	**72.7%**	**5.6%**	**0.0%**	**7.0%**
Rhinoceroses percentage positive samples from total (%)	0%	0%	0%	0%	0%
Zebras positive samples (number from zebra)	8	194	16	0	20
Rhinoceroses positive samples (number from rhinoceroses)	0	0	0	0	0
Zebras percentage positive samples from zebra (%)	**3.9**	**94.2**	**7.4**	**0.0**	**9.3**
Rhinoceroses percentage positive samples from rhinoceroses (%)	**0.0**	**0.0**	0.0	0.0	0.0
Kenya (number of total positive samples)	0	24	0	0	0
Tanzania (number of total positive samples)	8	170	16	0	20
Kenya (% of total positive samples)	**0.0**	**9.0**	**0.0**	**0.0**	**0.0**
Tanzania (%of total positive samples)	**3.0**	**63.7**	**5.6**	**0.0**	**7.0**
Location of positive samples	**7 Serengeti***	**160 Serengeti***	**12 Serengeti***		**all Serengeti***
Location of positive samples	**1 Ngorongoro****	**10 Ngorongoro****	**4 Ngorongoro****		

*The Serengeti National Park is located mainly in Tanzania but also in Kenia. Our samples were from Tanzania, but these wild animals can cross the border

**Ngorongoro is located in Tanzania
